# The effect of temperature conditions during growth on the transformation frequency of *Coccomyxa subellipsoidea C-169* obtained by electroporation

**DOI:** 10.1016/j.bbrep.2022.101220

**Published:** 2022-02-07

**Authors:** Kinga Kania, Anna Levytska, Anna Drożak, Borkowski Andrzej, Działak Paweł, Maksymilian Zienkiewicz

**Affiliations:** a Department of Molecular Plant Physiology, University of Warsaw, Warsaw, Poland; b Faculty of Geology, Geophysics and Environmental Protection, AGH University of Science and Technology, Krakow, Poland

**Keywords:** *Coccomyxa subellipsoidea* C-169, Electrotransformation, Stable genome transformation

## Abstract

In this study, we have shown that transformation efficiency of *Coccomyxa subellipsoidea* C-169 obtained by electroporation can be significantly increased by either supra- or sub-optimal growth temperatures.

## Highlights

• The algal cells size affects electroporation efficiency.
• Temperature during growth changes cells size.
• Fatty acids composition do not influence electroporation efficiency of algal cells.

## 1 Introduction

*Coccomyxasubellipsoidea* C-169 is a unicellular green alga adapted to the extremely difficult Antarctic environment (Holm-Hansen 1964). It is a psychrotolerant characterized by unique adaptation to cold and a wide range of temperature tolerance (from 0 °C to 30 °C and capable of surviving long periods of freezing) with an optimum growth temperature of around 20 °C [1]. One of the most interesting features of this alga that enables it to adapt to extreme cold conditions is the presence of a large variety of enzymes related to the synthesis and modification of lipids. This diversity of enzymes is the greatest among all the known sequenced chlorophytes [1]. This entails the possibility of synthesizing different membrane components. The presence of a variety of enzymes in this green alga and its metabolic properties may help to develop new technologies for converting lipids from microalgae into fuels or valuable fatty acids [2]. Recently, the utility of *C. subellipsoidea* C-169 as a potential biofuel producer has been intensively studied [[3], [4], [5]]. This prompted us to develop a system of transformation of *C. subellipsoidea* C-169 by electroporation [6]. Our previous research focused on finding the appropriate selection marker, determining the parameters of the electric field, and establishing the post-electroporation procedure for culturing the transformed cells.

Much research in the field of electrotransformation has focused on the optimization of parameters of electric pulses and chemical composition of the media used [[7], [8], [9]]. The correlation of the cell diameter with the level of electropermeabilization has also been investigated [10,11]. In general, the larger the cells, the more sensitive they are to the applied electric field intensity [[12], [13], [14]] Usually, there is a positive correlation between the percentage of electropermeabilized cells in a cell population and the increase in the field strength [14,15]. For example, an inverse correlation was reported between the intensity of the electric field and the cell diameter of protoplasts of *Vicia faba* [16]. A similar relationship was observed for the protoplasts of *Brassica napus* and *Brassica campestris* [17]. Another aspect of successful electropermeabilization is cell membrane fluidity. However, the conclusions of investigations on this aspect are inconsistent. A previous study reported that the fluidity of the cell membrane does not significantly affect reversible electroporation but may affect irreversible electroporation, which leads to cell death [18]. In contrast, other studies have reported that human lung cells could be more easily electropermeabilized as their membranes are less fluidic [19]. The temperature at which electroporation is performed significantly affects both the conductivity of the medium and the fluidity of the plasma membrane. For example, chilling from physiological temperature to 4 °C exerted a significant effect on cell membrane electropermeabilization of Chinese hamster lung fibroblasts and murine melanoma cells [9]. Moreover, in the case of the alga *Valonia utricularis*, the field strength needed for electropermeabilization increased significantly at lower temperature [16]. A similar observation was reported by other researchers [20]. In contrast, electropermeabilization of erythrocytes was not affected by temperature [21]. Many plant protoplast electroporation protocols recommend the use of ice for electroporation. Usually, after electroporation, protoplasts are incubated on ice [10]. The low temperature prolongs the duration of electro-pores formed on the protoplasts, which leads to increased DNA uptake [22]. Very few studies have investigated the effect of growth conditions of cells on the transformation frequency obtained by electroporation. One of such studies are those conducted on *Corynebacterium glutamicum* cells that could be easily transformed when grown in the presence of isonicotinic acid hydrazide and glycine [23]. This effect can be linked to the chemical influence on cells/electrical environments rather than to environmental factors such as temperature or light conditions during growth. In the present study, our objective was to investigate the possibility that sub-optimal growth conditions may modulate the electrotransformation efficiency of *C. subellipsoidea* C-169. On the basis of the obtained results, we show that simple physical modulation of growth conditions can significantly increase the efficiency of this process.

## 2 Materials and methods

### 2.1 Cell cultures

*C. subellipsoidea* C-169 strain #NIES 2166 was derived from Microbial Culture Collection, National Institute for Environmental Studies, Japan.

Cells of C-169 used in the next step for electrotransformation were cultivated 15 days in liquid Bold's Basal Medium (BBM) with constant shaking at four different temperature regimes: 4 °C, 10 °C, 20 °C and 27 °C under continuous white light at intensity 35 μmol photon m^−2^s^−1^.

Cells of C-169 after electroporation were cultivated in liquid BBM and further on Petri dishes filled with BBM medium, solidified by addition of 1% agar (Basica LE, Prona, EU) at 20 °C under white light, photoperiod (12/24, light intensity 35 μmol photon m^−2^s^−1^). The medium was supplemented with 40 μg/ml hygromycin B (Pan ReacAppliChem ITW Reagents) for the selection of transformed cells.

*Escherichia coli* strain DH5α were used for maintenance of transformation vector. Bacterial cells were cultured in liquid LB medium at 37 °C or on Petri dishes with LB medium solidified by addition of 1% agar. For the selection of transformed cells, the medium was supplemented with kanamycin (30 μg/ml).

### 2.2 Plasmid used in this work

Plasmid pCAMBIA1391Z (11 227 bp) containing *hpt*II gene coding for hygromycin B phosphotransferase which is expressed in plants thanks to strong cauliflower mosaic virus CaMV35S promoter (https://cambia.org) was used for transformation.

### 2.3 Electrotransformation

Electrotransformation of C-169 cells was performed following an adaptation of a previously described procedure with some modifications [6], using a GenePulser apparatus (Bio-Rad Laboratories Limited, UK). Briefly, for transformation cells were washed with ice cold H_2_O three times and resuspended in ice cold H_2_O in final concentration of 1 × 5 × 10^8^ cells mL^−1^. The number of cells of C-169 from cultures growing at 4 °C, 10 °C, 20 °C and 27 °C has been verified every time by counting with the use of a hemocytometer. The 10 μg of plasmid DNA and a 10-fold excess of salmon sperm DNA (Invitrogen, http://www.invitrogen.com) was supplied into the 2 mm or 4 mm electroporation cuvette containing 0.2 ml of C-169 cultures. Electroporation was conducted at 25 μF and 200 or 600 Ω (Ω) with applied voltage 1900 V or 2750 V. Electric field strength E was 9.5 kVcm^−1^ or 6.8 kVcm^−1^ as E = V/d where V is the applied voltage in volts and d is the distance between the electrodes in centimetres. Cuvettes with C-169 cells were incubated on ice for 5 min directly after electroporation, and then cells were transferred to a 10 ml fresh BBM medium in 25 cm^3^ tissue culture flasks. The cells were constantly shaking and incubated by 24 h in the dark at 20 °C, and then they were moved to the light. Algal cultures were grown under white light photoperiod, as described in the “Cell cultures” section. After 3 days from transformation, green alga cultures were supplemented with 40 μg/ml hygromycin B and grown in liquid BBM medium by the next 14 days. Then, cells were harvested by centrifugation at 2000*g*, resuspended in 200 μl of BBM, and spread onto agar plates supplemented with hygromycin B at final concentration of 40 μg/ml. After 6 weeks of growth on the agar plates, colonies were scored. All results are presented as mean values ± standard error (SE). The significant differences between means were determined using Student's *t*-test for paired observations at a confidence level of α = 0.05.

### 2.4 Identification of a hygromycin B-resistant mutants of C-169

Randomly chosen hygromycin B resistant colonies has been tested for the presence of *hpt*II gene coding for hygromycin B phosphotransferase via standard PCR technique with use of primers hptIIif 5′ AAC TGC CCG CTG TTC TAC AAC C 3′ and hptIIir 5′ TGC TGC TCC ATA CAA GCC AAC C 3.

### 2.5 Cell counting and size measurements

Cells were observed using the microscope Olympus BX43F (Tokyo, Japan). The number of algal cells were counted with the use of hemocytometer (Tiefe Depth Profondeur 0.1 mm, Superior Marienfeld, Germany) according to manufacturer's protocol. The sizes of C-169 cells were measured with the use of Olympus cellSens Standard software. The size of cells was expressed as the longest diameter of the oval cells. The average of 50 cells was measured per each condition and the data is presented as the mean ± standard deviation.

### 2.6 Analysis of fatty acids

Fatty acid composition was analysed as follows. Lyophilized and previously weighed material (cells or thylakoids) was placed in 1.5 ml Eppendorf tube and 0.5 ml 1 M KOH in methanol was added. Saponification was conducted for 30 min at 60 °C. Then derivatization of fatty acids to methyl esters (FAME) was carried out using 2.5 ml of 3% H_2_SO_4_ in methanol (1 h at 60 °C) added directly to the mixture after saponification. Subsequently, 2 ml of hexane was added to extract the FAME. After extraction, the nonpolar organic phase was dried by anhydrous Na_2_SO_4_ and neutralized by CaCO_3_. The obtained sample was analysed by GC-MS (Agilent 7890A GC z 5975C MSD). The temperature program was: 110 °C for 3 min, 3 °C min^−1^ to 200 °C for 3 min, then 2 °C min^−1^ to 250 °C for 14 min. Gas flow (He) 1.8 ml min^−1^. The analysis was conducted with 60 m column (DB-5MS, 250 μm, 0.25 μm), using bacterial acid methyl esters (BAME mix, 47080-U, Sigma-Aldrich) and 37 component FAME mix (CRM47885, Supelco) as a standard. All results are presented as mean values ± standard error (SE). The significant differences between means were determined using Student's *t*-test for paired observations at a confidence level of α = 0.05.

## 3 Results and discussion

**Fig. 1:**
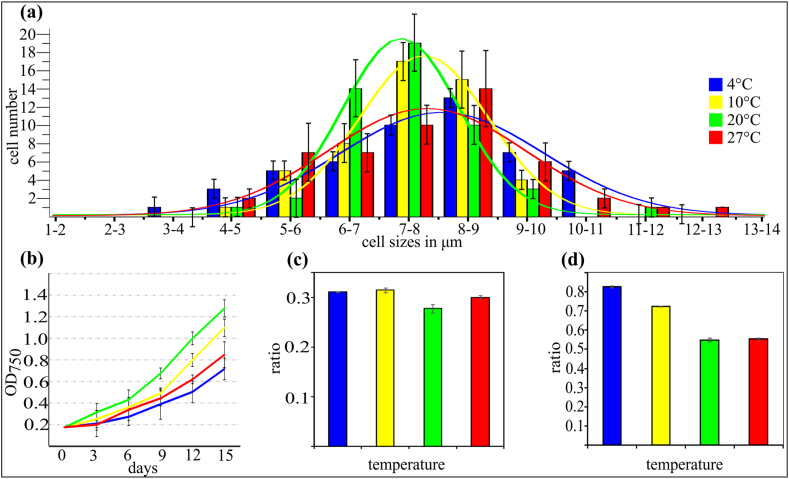
Morphological and physiological adaptation of C-169 cells cultured in four different temperature regimes. (**a)** Histogram showing cells size distribution for randomly chosen 50 cells taken from 4-,10-,20- and 27**°**C**. (b)** Growth dynamics of algal cells cultured for fifteen days in 4-,10-,20- and 27**°**C. **(c)** The ratio of the total saturated (SFA) to unsaturated (PUFA and MUFA) fatty acids **(d)** The ratio of C16 fatty acids to C18 fatty acids. Mean ± SE (n = 3).

Table 1 presents the transformation efficiency of C-169 taken from four temperature regimes. Two field strengths were tested to determine whether the transformation efficiency correlates positively with the increase in the field strength. The transformation efficiency was ∼1/3 higher at 9.5 kV cm^−1^ than that at 6.8 kV cm^−1^. Interestingly, the number of colonies resistant to hygromycin B was comparable for cultures taken from 4 °C to 27 °C, and it was ∼3 times higher than that for cultures from physiological optimum temperatures of 20 °C and 10 °C. The successful introduction of the *hpt*II gene, coding for hygromycin B phosphotransferase, into the genomes of C-169 transformants was verified in all hygromycin B-resistant randomly tested cells. The average C-169 cell size, measured from four different temperature regimes, is presented in Table 2. In general, the cell size was slightly larger for cells taken from 4 °C to 27 °C than that for cells taken from 20 °C to 10 °C. However, because the statistical analysis showed α > 0.05, the observed differences were not statistically significant. In fact, the average size of C-169 cells did not fully represent the populations of algal cells at different temperature regimes. Fig. 1a presents a histogram of algal cells grouped according to their sizes. The algal cultures from 4 °C to 27 °C contained significantly larger sub-populations of cells with sizes of ≥9 μm, although the growth dynamics of these two temperature variants were lower than that of the variants growing at optimum temperature (Fig. 1b). The highest growth rate was noted at 20 °C, a slightly lower growth rate at 10 °C, and the lowest growth rate at 4 °C and 27 °C. The typical size of cells at all temperatures was calculated as the maximum in all classes of gaussian distribution. The F-test showed that cells grown at 10 °C and 20 °C were smaller than those grown at 4 °C (p < 0.05); however, the size of the cells grown at 27 °C was not significantly different from that of the cells grown at 4 °C (p > 0.05).

**Table 1:** The transformation efficiency of C-169 cultured in four different temperature regimes.

Field strength E = V/d	Number of colonies resistant to hygromycin B^(^*^)^			
	4 °C	10 °C	20 °C	27 °C
9,5 kVcm^−1^	69 ± 22	13 ± 6	21 ± 7	58 ± 26
6,8 kVcm^−1^	49 ± 13	7 ± 4	14 ± 9	36 ± 11

Cells were electroporated, and then, they were grown initially in liquid medium supplemented with hygB (40 μg/ml) and then on agar plates with *hyg*B, as described in the “Materials and methods” section. The colonies were scored after 6 weeks of growth on plates. Samples electroporated in the absence of pCAMBIA1391Z did not give any resistant colonies.

^(^*^)^ Mean ± SE (n = 3). Student's *t*-test revealed that differences were statistically significant.

**Table 2:** The average sizes of C-169 cells cultured in four different temperature regimes.

Temperature	Size
4 °C	7,68 μm ± 1,68
10 °C	7,26 μm ± 1,16
20 °C	7,27 μm ± 1,18
27 °C	7,83 μm ± 1,76

^(^*^)^ Mean ± SE (n = 3).

One of the main adaptation mechanisms of algal cells to different temperatures is modulation of their membrane fluidity by changing fatty acid composition [24,25], for review see Ref. [26]. Changes in membrane fluidity affected by the modulation of fatty acid length have also been observed [27,28]. Indeed, fatty acid composition was affected by temperature in C-169 algal cells. While the ratio of the sum of saturated fatty acids (SFA) to the sum of unsaturated fatty acids (PUFA and MUFA), as shown in Fig. 1c, was approximately the same for all the tested growth temperatures, the relationships between the most abundant C16 and C18 fatty acids in cells, shown in Fig. 1d, indicated that membrane fluidity in C-169 cells is modulated mainly by the change in the length of fatty acid pool rather than the level of unsaturated fatty acids.

Temperature is the major physical factor affecting algal growth and cell size [29,30]. For example, an inverse correlation was reported between temperature and the cell size of plankton [31]. Temperature stress usually negatively affects the growth rate and alters the shape of algal cells [32]. A slow growth rate was observed for C-169 cells grown at 4 °C and 27 °C, indicating that both temperatures are sub-optimal/stressful. The differences in the average cell size of transformed C-169 cells measured for optimal and sub-optimal temperature regimes were not statistically significant and could not satisfactorily explain almost three times higher transformation efficiency for C-169 cells taken from sub-optimal conditions. The situation, however, changes if we analyze the histogram of algal cells grouped by their sizes as shown in Fig. 1a. This indicates that the reason for higher transformation efficiency of C-169 cells is the significant increase in the sub-population of cells of size ≥9 μm in the sub-optimal/stressful environmental conditions. This explanation is supported by findings of other researchers who reported that significant differences in electroporation efficiency were correlated with mesophyll protoplast size in turnips [17]. As expected, the analysis of fatty acid composition of C-169 algal cells confirmed the modulation of membrane fluidity in response to diverse temperature regimes. The electroporation procedure was performed on ice with previously chilled C-169 cultures. In these conditions, the membrane fluidity is expected to be the highest in C-169 cells adapted to 4 °C and the smallest for cells grown at 27 °C. Unexpectedly, we observed similar electroporation efficiency for C-169 cells taken from these two extreme temperature conditions. This finding suggests that the presence of a cell sub-population of ≥9 μm in size, rather than the overall fluidity of the membrane, is the key factor that influences the transformation efficiency of C-169 cells. However, a combination analysis of factors responsible for electroporation efficiency is needed for more clear understanding of the effect of each factor. The size of C-169 cells was affected by low and high temperatures, but most probably, a similar effect could be achieved by the modulation of light intensity as observed for *Sinapis alba* [33] or *Dunaliella salina* cells [34].

Here, we show for the first time that it is possible to increase the electrotransformation efficiency by simple modulation of growth conditions of C-169 cells that change the cell size. The results indicate that the use of optimal growth conditions for transformed cells may not always be the best approach for successful, efficient electrotransformation. We believe that the application of sub-optimal or even stressful conditions may help to successfully transform the cells of other organisms.

## Funding

This research did not receive any specific grant from funding agencies in the public, commercial, or not-for-profit sectors.

## Declaration of competing interest

We do not report any existing or pending conflict of interest regarding this manuscript.

## Data Availability

The authors do not have permission to share data.
